# Comprehensive Assessment of Collagen/Sodium Alginate-Based Sponges as Hemostatic Dressings

**DOI:** 10.3390/molecules29132999

**Published:** 2024-06-24

**Authors:** Leilei Sun, Yanyan Shen, Mingbo Li, Qiuting Wang, Ruimin Li, Shunmin Gong

**Affiliations:** College of Life Science, Yantai University, Yantai 264005, China; yyshenqx1999@163.com (Y.S.); sllshd1991@163.com (M.L.); wqt1981979471@163.com (Q.W.); 17861135192@163.com (R.L.); gong1394@foxmail.com (S.G.)

**Keywords:** collagen, sodium alginate, crosslinking, biocompatibility, hemostatic dressing

## Abstract

In our search for a biocompatible composite hemostatic dressing, we focused on the design of a novel biomaterial composed of two natural biological components, collagen and sodium alginate (SA), cross-linked using 1-ethyl-3-(3-dimethylaminopropyl) carbodiimide/N-hydroxysuccinimide (EDC/NHS) and oxidized sodium alginate (OSA). We conducted a series of tests to evaluate the physicochemical properties, acute systemic toxicity, skin irritation, intradermal reaction, sensitization, cytotoxicity, and in vivo femoral artery hemorrhage model. The results demonstrated the excellent biocompatibility of the collagen/sodium alginate (C/SA)-based dressings before and after crosslinking. Specifically, the femoral artery hemorrhage model revealed a significantly shortened hemostasis time of 132.5 ± 12.82 s for the EDC/NHS cross-linked dressings compared to the gauze in the blank group (hemostasis time of 251.43 ± 10.69 s). These findings indicated that C/SA-based dressings exhibited both good biocompatibility and a significant hemostatic effect, making them suitable for biomedical applications.

## 1. Introduction

Hemorrhage is a common phenomenon in daily life, especially in traffic accidents and uncontrolled arterial injury hemorrhage. It poses a significant threat to life and is the primary cause of injury and death. Therefore, effective hemostatic materials are required for timely treatment [1,2,3].

Over the past few decades, various hemostatic dressings have been developed with different levels of efficacy, but none are considered perfect. An ideal hemostatic dressing should ensure a moist wound environment, absorb excess wound exudate, provide rapid and sustainable hemostatic efficacy, be non-cytotoxic and breathable, and prevent bacterial invasion. It should also support processes such as wound re-epithelialization [4,5]. Recently, there has been an increasing use of commonly employed hemostatic materials, including chitosan [6], collagen [7], gelatin [8,9], zeolite [10], sodium alginate (SA) [11], oxidized regenerated cellulose [12], and cyanoacrylate [13]. These biomaterials, whether of plant or animal origin, exhibit excellent biocompatibility and accelerate tissue reconstruction capabilities [14]. Collagen, a highly abundant functional protein in the body, exhibits a strong affinity and compatibility with protein molecules on the surface of skin. It also possesses high biodegradation safety [15,16]. When used in the form of a sponge, collagen provides a 3D framework and support for cell growth. It acts as a riveting and supporting agent for cells, while also playing a crucial role in behavioral processes such as cell attachment, spreading, and proliferation [17,18,19,20]. Furthermore, collagen sponges have been reported to enhance hemostatic performance, encourage platelet attachment and clustering, and release coagulation components to facilitate blood coagulation [7,21]. Due to these properties, collagen-based materials have found extensive use in various biomedical applications [22]. Another important component, SA, is a polysaccharide found in the cell walls of brown algae (commonly known as brown seaweed). SA contributes to the pliable and strong structure of brown algae [23,24]. It exhibits good biocompatibility and histocompatibility, is naturally non-toxic, has a high level of water content, and possesses certain hemostatic properties [25,26]. SA dressings have the capability to form gels through crosslinking with calcium and magnesium ions in solution. They can also be processed into lyophilized porous tablets in the form of foams or fibrous dressings [27,28,29]. These dressings effectively absorb exudates in a dry form and can be removed from the wound site without causing significant tissue damage. Additionally, they protect the wound from maceration, enhance gas exchange, and establish a physiologically moist environment, which helps minimize bacterial infection and promotes rapid re-epithelialization and granulation tissue formation [30,31]. Numerous studies have demonstrated the efficacy of these dressings [32].

Collagen can be extracted from the tissues of any animal. However, the use of animal-derived collagen may lead to complications such as allergic reactions and the spread of pathogens, and there may be religious prohibitions [33]. On the other hand, extracting collagen from marine organisms offers a safer and more economical alternative method [34,35]. Nile tilapia, an economically significant fish worldwide, provides an opportunity to extract collagen from its by-products, specifically the fish skin. This approach avoids the risks associated with land-based sources, such as diseases and religious concerns, while also minimizing resource waste and environmental pollution [36,37].

The limited physical and chemical properties of pure collagen dressings often lead to disadvantages such as weak mechanical strength, easy breakage, and difficulty in stopping bleeding from large wounds. Pure sodium alginate exhibits low mechanical strength due to its high water solubility, resulting in inadequate adhesion sites for cells [38,39,40]. These issues can be addressed through crosslinking and the blend of collagen and sodium alginate, which can be achieved through physical, chemical, or enzymatic methods [41,42,43,44,45]. Jin et al. [46] developed a composite microsphere (CSCM) incorporating three natural ingredients: carboxymethyl chitosan, SA, and collagen. The study findings demonstrated that CSCM accelerated coagulation and wound closure, with the benefit of natural material degradation in vivo, despite some intradermal irritation from the CSCM material. Huang et al. [47] produced a biodegradable powdered hemostatic composite (SACC) through emulsification and crosslinking methods, leveraging SA, carboxymethyl chitosan (CMC), and collagen. The hemostatic material exhibited a uniform size distribution, a textured surface, and effective water absorption. Through various tests and models, SACC exhibited superior hemostatic properties, biodegradability, and biocompatibility for clinical applications. Glutaraldehyde was a widely used chemical cross-linker, but its cytotoxicity has raised concerns [48]. EDC/NHS-induced crosslinking involves a condensation reaction without the introduction of a chemical reagent. This type of crosslinking, known as “zero-length” crosslinking, is generally considered to be biocompatible and non-toxic [49]. EDC/NHS has also been explored as a potential alternative to glutaraldehyde. Additionally, SA can serve as a crosslinking agent to stabilize collagen. In the presence of NaIO4, the hydroxyl group adjacent to SA can be selectively oxidized to two highly reactive aldehyde groups. Therefore, oxidized sodium alginate (OSA) can be used as a crosslinking agent for proteins, peptides, and other biologically active substances. Composite scaffolds combining collagen and alginate have been extensively studied for biomedical applications. These scaffolds not only improved the mechanical properties but also promoted the inflammatory phase of wound healing [41]. However, there is limited research on the effect of crosslinking on the biological properties of collagen-based biomedical materials, apart from their impact on the mechanical properties and biodegradability.

This study aims to prepare collagen/sodium alginate (C/SA) dressings by mixing collagen and SA. The C/SA dressings were then cross-linked using EDC/NHS. Additionally, SA was oxidatively modified to obtain polyaldehyde OSA, which was further cross-linked to the collagen dressings to obtain C/OSA sponges. The physicochemical and biological properties, hemostatic activity, and biocompatibility of the C/SA-based dressings were systematically investigated. The effect of crosslinking on these properties was also explored. The findings of this study have the potential to broaden the application of collagen and SA in medical hemostatic therapy and provide new insights for the development of C/SA-based hemostatic dressings.

## 2. Results and Discussion

### 2.1. Physicochemical Properties of C/SA-Based Dressings

**Figure 1:**
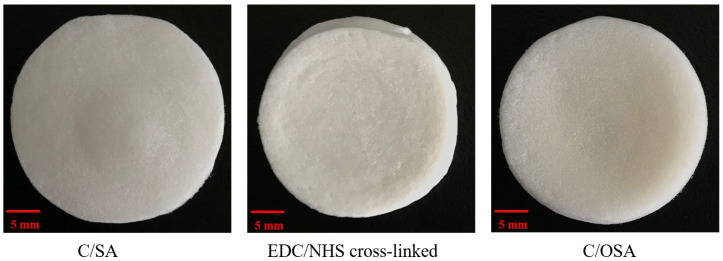
Macro-morphology of C/SA-based dressings.

The macro-morphology of the three C/SA-based dressings after lyophilization was observed in Figure 1. The diameter of the three C/SA-based dressings was 22 mm. The surface of the C/OSA dressing appeared yellowish compared to C/SA and EDC/NHC cross-linked dressings. All three dressings exhibited a three-dimensional pore structure, facilitating rapid absorption of exudate and water from the wound. Additionally, these structures provided attachment points for platelets, promoting thrombus formation and facilitating coagulation. Therefore, C/SA-based dressings have the potential to be effective in blood absorption and can be considered for use as hemostatic dressings. 

Table 1 presents the measurements concerning loss on drying, total ash content, and carbodiimide residue. Both C/SA and C/OSA exhibited a loss on drying exceeding 15% and a total ash content surpassing 2%. Nevertheless, the EDC/NHS cross-linked C/SA dressing demonstrated a loss on drying below 15% and a total ash of less than 2%, thus meeting the criteria for collagen dressings within the biomedical materials domain. Additionally, no carbodiimide residue was detected in the EDC/NHS cross-linked C/SA dressing. In our previous research, dressings treated with EDC/NHS and OSA demonstrated good solution stability and hygroscopicity, with values of 25.93 ± 0.56 and 25.12 ± 0.43, respectively [50]. The crosslinking of EDC/NHS and OSA resulted in a shift in the amide SA peak from 3315 cm^−1^ to approximately 3282 cm^−1^ [50]. The stretching peaks of the O-H and N-H bonds were notably enhanced [50]. OSA displayed a characteristic vibrational peak of the aldehyde group at 1615 cm^−1^, and the C/OSA dressing exhibited an absorption peak of the Schiff base structure at 1653 cm^−1^ [50]. The successful introduction of the carboxyl group was evidenced by the disappearance of the absorption peak at 1417 cm^−1^ and the appearance of two new absorption peaks at 1445 cm^−1^ and 1409 cm^−1^ [50]. In terms of SEM analysis, the cross-linked sponges and unmodified sponges showed structural similarity and were characterized by their porous nature, which facilitated cell growth [50].

### 2.2. Biocompatibility of C/SA-Based Dressings

The biocompatibility of the materials was assessed through various tests including an acute systemic toxicity assay, an irritation test, a sensitization test, and a cytotoxicity assay. 

**Figure 2:**
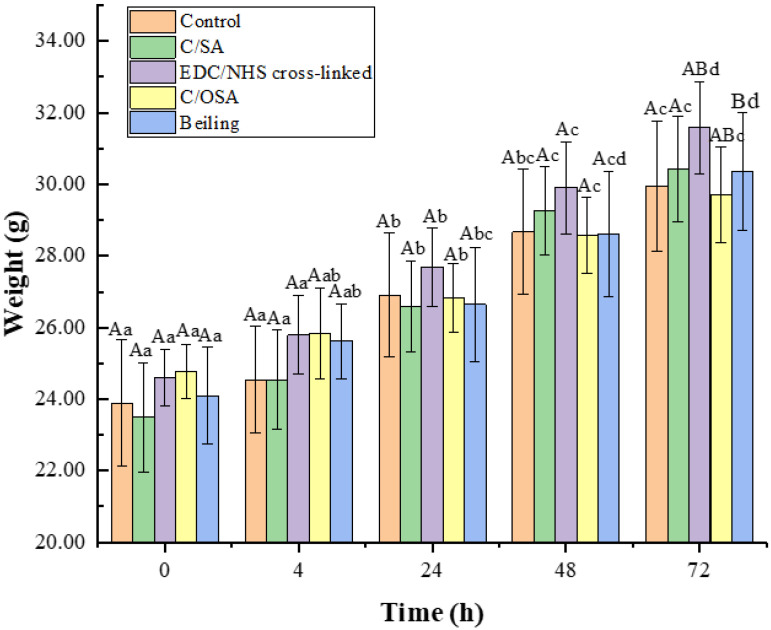
Weight changes in mice. Different capital letters in the same group represent significant difference, and different lowercase letters in different groups represent significant differences (*p* < 0.05).

The evaluation of potential harmful effects resulting from biomedical materials or their leach liquor being absorbed by an organism and localized to a specific body part is typically carried out using the acute systemic toxicity assay. In this study, high doses of tail intravenous injections in KM mice were used to comprehensively assess acute systemic toxicity. As displayed in Figure 2, mice gained weight during the observation period. Neither the control group nor the C/SA-based dressing group of mice showed adverse effects such as slowed movement or reduced feeding during the following 72 h. All mice exhibited sensitivity to sound, light, and other stimuli and showed no signs of breathlessness, tearing, or oedema. Additionally, the mortality rate was 0%. Therefore, it could be concluded that the C/SA, EDC/NHS cross-linked C/SA, and C/OSA had no acute systemic toxicity. 

To ensure the safety of applying novel biomedical materials to humans, it is crucial to evaluate their potential for skin irritation [51]. Hence, a dermal irritation test and an intradermal reaction test were used in New Zealand white rabbits to evaluate the local skin irritation effects of C/SA-based dressings. No deviations from the expected normal state were observed in any of the test animals throughout the study. The results (Table 2) indicated that none of the C/SA, EDC/NHS cross-linked C/SA, C/OSA, Beiling, or gauze samples exhibited any pathological reaction, such as erythema, scar formation, and edema, nor did they cause any unusual clinical symptoms. The PIIs were all calculated to be zero. Therefore, the C/SA-based dressings met the criteria for biomaterial irritation tests. 

The assessment of the allergic response potential of the C/SA-based dressings is also presented in Table 2. Skin changes on the guinea pigs were observed for 24 h, 48 h, and 72 h after the patch test. No skin sensitization was observed on the skin surface of any guinea pigs at any time point. Both the control and experimental groups exhibited a sensitization score of zero, consistent with findings in the literature indicating that atelocollagen biomaterials lacking the peptide region at the antigenic end demonstrate good biocompatibility [52,53]. Therefore, the C/SA-based dressings were determined to be non-sensitizers in the sensitization test and exhibited good biocompatibility.

**Figure 3:**
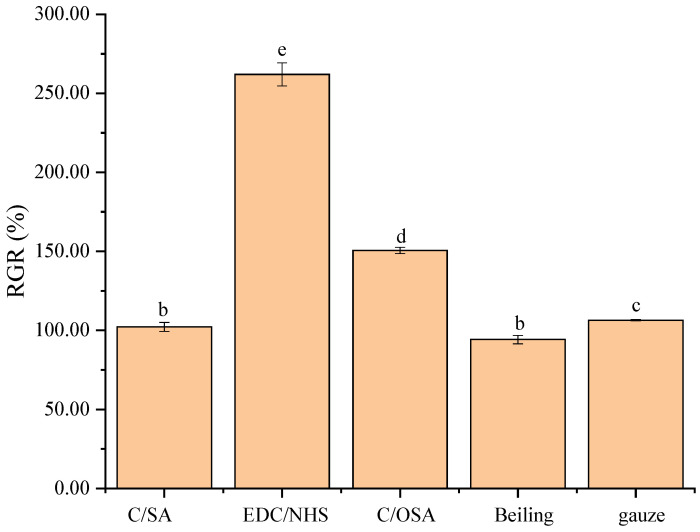
Relative growth rate of C/SA-based dressings. Different lowercase letters in different groups represent significant differences (*p* < 0.05).

Cytotoxicity is a crucial parameter to consider when studying hemostatic materials. Fibroblasts, which promote angiogenesis, play a significant role in this regard. Therefore, it is important to study the cytotoxicity of fibroblasts when evaluating hemostatic materials. The MTT method was used to detect cytotoxicity and was considered an essential pre-screening method due to its high sensitivity and convenience [54,55]. In Figure 3, the percentages of RGR were 102.19%, 261.99%, 150.61%, 94.23%, and 106.41%, and all the samples were found to be non-cytotoxic to L929 cells within 48 h. All the C/SA-based dressings prepared had a proliferation rate of >100% compared to commercial Beiling and met the non-toxicity criteria of ISO 10993 [56]. Moreover, the EDC/NHS cross-linked dressings exhibited higher cytocompatibility, probably due to the formation of additional amides after crosslinking, which modulate the affinity of the dressing for cell growth [57]. There was also a significant difference between the C/OSA and EDC/NHS cross-linked dressings, potentially because the materials contained aldehyde groups that reacted with intracellular proteins and polysaccharides, thereby affecting cell proliferation. However, the cytotoxicity of the dressings in each group was still graded as 0 according to the cytotoxicity criteria. The cytotoxicity tests have demonstrated that the C/SA-based dressings had a positive effect on cell proliferation and differentiation, indicating excellent cytocompatibility and biosafety.

### 2.3. Hemostatic Evaluation In Vivo

Acute blood loss resulting from aortic injury is a significant threat to human life. The rat femoral artery bleeding model offers a more accurate simulation of acute blood loss in the human body. As such, this study aimed to construct a rat femoral artery bleeding model to assess the in vivo hemostatic efficacy of the developed hemostatic materials [58]. The results in Table 3 present the bleeding time and blood loss associated with the use of C/SA-based dressings in the femoral artery hemorrhage model. In the blank control gauze group, it took an average of 251.43 ± 10.69 s to achieve hemostasis, and the total blood loss was 1.78 ± 0.08 g. In comparison, the C/SA-based dressings demonstrated significant advantages in the rat femoral artery model, with shorter hemostasis time and lower blood loss than the control group and the single collagen material (180 ± 16 s, 1.38 ± 0.08 g) [59]. These positive outcomes could be attributed to the porous structure of the dressings [60]. The porous structure allowed for high absorption of water from the blood, which synergistically promoted blood coagulation. Additionally, the porous structure facilitated platelet adhesion to the dressing’s pore wall, further enhancing hemostasis [61,62]. The presence of free hydroxyl groups also contributed to improved hemostasis by enabling better platelet function [63]. Overall, the prepared C/SA-based dressings effectively stopped bleeding and reduced the risk of infection throughout the experimental period. This study highlights the potential for future clinical applications of C/SA-based dressings in spinal cord injury and liver tissue engineering.

**Figure 4:**
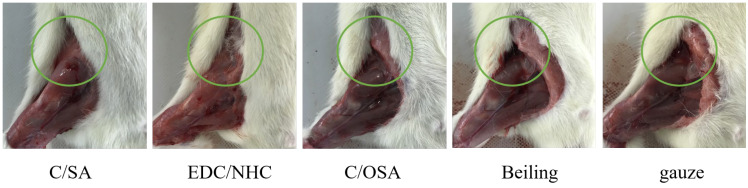
Diagram illustrating the femoral artery hemorrhage model post-hemostasis.

Figure 4 illustrates the conditions following hemostasis. Upon contact with the wound, the dressings absorbed a substantial amount of blood, leading to coagulation on its surface and at various bleeding points. This effectively stopped further blood loss from the wound. However, the gauze did not absorb the blood quickly enough, causing it to flow around the wound. No significant differences were observed in terms of bleeding time and blood loss between the C/SA and C/OSA groups. Beiling also resulted in clot formation due to slower absorption compared to C/SA-based dressings. In general, C/SA-based dressings provide a larger surface area and better mimic the structure of the extracellular matrix (ECM) compared to gauze. Therefore, they could make closer contact with the blood in the wound, facilitating rapid hemostasis. However, further research is needed to explore methods for improving the adhesion of C/SA-based dressings.

## 3. Materials and Methods

### 3.1. Reagents and Experimental Materials 

Nile tilapia skin collagen was provided by our laboratory [64]. 

MTT was purchased from Sigma-Aldrich Co., Ltd. (St. Louis, MO, USA). HEPES was acquired from Thermo Fisher Scientific Co., Ltd. (Burlington, MA, USA). DMEM, FBS, and trypsin were provided by Gibco Co., Ltd. (Grand Island, NY, USA). The study utilized reagents of analytical grade.

Male Wistar rats (seven weeks old, weighing 200–220 g) and male KM mice (six weeks old, weighing 18–20 g) were acquired from Jinan Pengyue Laboratory Animal Co., Ltd. (Jinan, China). Male Guinea pigs (seven weeks old, weighing 300–350 g) and male New Zealand white rabbits (eighteen weeks old, weighing 2.0–2.5 kg) were purchased from Jinan Jinfeng Laboratory Animal Co., Ltd. (Jinan, China). The study protocol received approval from the Ethical Committee of Yantai University (Approval Code: 20230521s145; Approval Date: 21 May 2023).

### 3.2. Fabrication of C/SA-Based Dressings

#### 3.2.1. Fabrication of C/SA Dressings

C/SA dressings were prepared using a modified version of our previously reported method [59]. Collagen was mixed with 0.5 M acetic acid while stirring into a 10 mg/mL solution. The solution was then dialyzed with 20 times the volume of deionized water and stirred at 100 rpm using a magnetic stirrer. Additionally, a 10 mg/mL of SA solution was prepared using deionized water. The SA solution was gradually added to the collagen solution in a 1:5 ratio while stirring at 150 rpm with a magnetic stirrer. The resulting mixture was then centrifuged at 4 °C for 8 min at 8000 rpm. Following this, 10 mL of the post-defoaming solution was dispensed into a six-well cell culture plate and initially frozen at −40 °C for 12 h. Afterwards, the resulting mixture was subjected to vacuum freeze-drying at a temperature of −60 °C and a pressure of 0.06 mbar for a duration of 48 h. The dressings were then sterilized by ^60^Co for 18 h with an accumulative dose of 20 kGy.

#### 3.2.2. Fabrication of EDC/NHS Cross-Linked C/SA Dressings

**Figure 5:**
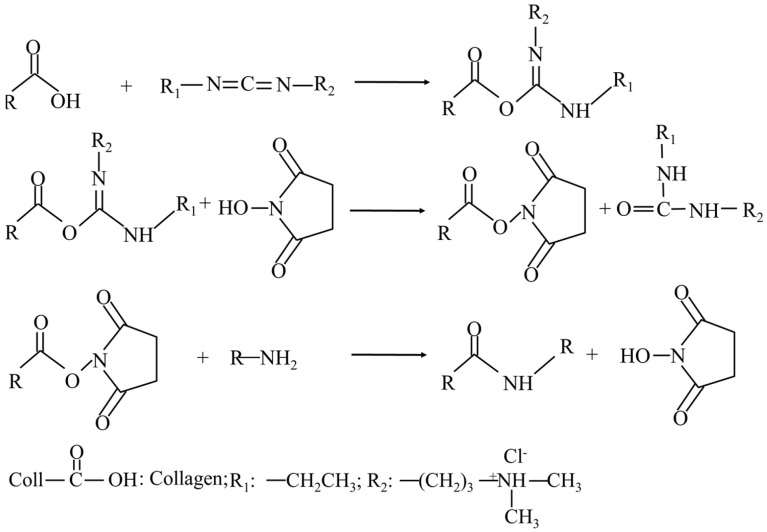
Schematic for the formation of crosslinking of EDC/NHS.

A 40% ethanol solution with 50 mM MES buffer was prepared. The C/SA dressings were immersed in this solution for 30 min. Subsequently, the samples were transferred to a solution consisting of 40% ethanol, 50 mM MES buffer (pH 5.5), and EDC and NHS in a ratio of 2.5:1, with an EDC concentration of 100 mM. Crosslinking was carried out for 4 h at 25 °C. Subsequently, the dressings underwent two washes with 0.1 M Na_2_HPO_4_ and were then rinsed with 1, 2, and 4 M NaCl overnight. Following repeated washing with deionized water, the dressings underwent another round of lyophilization. The dressings were then sterilized by ^60^Co for 18 h with an accumulative dose of 20 kGy. The process of the EDC crosslinking reaction is illustrated in Figure 5, where EDC initially reacted with the carboxyl group to create an O-acylurea intermediate. This activated intermediate then bonded with the amino group, forming a complex through amide crosslinking. However, this intermediate was susceptible to hydrolysis in aqueous solutions. The addition of NHS facilitated the formation of a more stable ester intermediate with the EDC-activated carboxyl group, ultimately enhancing the efficiency of the EDC-mediated coupling reaction. MES functioned as a buffer solution, playing a role in regulating the pH within the reaction environment.

#### 3.2.3. Fabrication of OSA and C/OSA Dressings

To prepare solution A, 10 g of SA was weighed and dissolved in 50 mL of ethanol. Similarly, for solution B, 10 g of NaIO_4_ was dissolved in 125 mL of deionized water. Solution A and solution B were mixed and stirred at 25 °C for 8 h to oxidize, while being protected from light. The oxidation reaction was terminated through adding ethylene glycol (2.6 mL) in the same molar quantity as sodium periodate and letting it react for approximately 30 min. Next, the reacted mixture was added to anhydrous ethanol with vigorous stirring using an electric blender for more than 30 min to precipitate a solid. The ratio of the volume of the reaction mixture to anhydrous ethanol should be 1:5. After suction filtration, the resulting precipitate was then dissolved in 500 mL of deionized water, followed by precipitation in ethanol. Subsequently, the resulting precipitate was dissolved in deionized water and dialyzed with 20 times the volume of deionized water, stirring at 100 rpm using a magnetic stirrer for 3 d to remove small molecules including NaIO_4_ and C_2_H_6_O_2_ that did not participate in the reaction. After precipitation with ethanol, the mixture was filtered and positioned in a flat container and subjected to vacuum freeze-drying at a temperature of −60 °C and a pressure of 0.06 mbar for a duration of 48 h to produce OSA.

For the formation of solution C, collagen was dissolved with 0.5 M HAc to prepare a solution with a concentration of 10 mg/mL. Similarly, lyophilized OSA was mixed with deionized water to form a 10 mg/mL solution D. Solution D was then slowly added to solution C in a ratio of 1:5 with high-speed stirring, and the resulting mixture was cross-linked at 4 °C for 24 h. Air bubbles were subsequently removed through centrifugation at 4 °C for 8 min at a speed of 8000 rpm. The resulting mixture was then injected into a 6-well plate with approximately 10 mL. The plate was preliminarily frozen at −40 °C for 12 h and then lyophilized to obtain the C/OSA dressings. The dressings were then sterilized by ^60^Co for 18 h with an accumulative dose of 20 kGy.

### 3.3. Physicochemical Properties of C/SA-Based Dressings

According to the China Pharmacopeia 2015, fourth section [65], standard procedures were followed to determine the loss on drying, total ash, and carbodiimide residue.

### 3.4. Biocompatibility Evaluation

The biocompatibility evaluation of the C/SA-based dressings was conducted in accordance with the ISO standard.

#### 3.4.1. Acute Systemic Toxicity Assay

The acute systemic toxicity of C/SA-based dressings was evaluated using the United States Pharmacopoeia, National Formulary, In Vivo Biological Reactivity Test [66]. To perform the experiment, 1 × 1 cm samples of the C/SA-based dressing with a thickness of 0.5 mm were prepared. The leaching solution was prepared by maintaining a proportional ratio of 3 cm^2^ total surface area of the materials per mL of normal saline. These samples were then immersed at 37 °C for 72 h. A total of forty-five KM mice were randomly assigned to five groups. The blank control group received an injection of saline (50 mL/kg body weight) via the cauda vein. The remaining four experimental groups received injections of the leach liquor from C/SA, EDC/NHS cross-linked, C/OSA, and commercial collagen dressings, respectively. The general conditions of the animals, including muscle movement, body weight, respiration, mortality, and clinical signs such as oedema and erythema, were carefully observed at specific time points (4, 24, 48, and 72 h). The level of toxicity was categorized into four grades based on different symptomatic manifestations, such as none, mild, moderate, and severe. The C/SA-based dressings were considered to meet the requirements of this test if animals treated with the C/SA-based dressings did not exhibit a significantly higher biological response compared to animals treated with the blank control over the 72 h observation period. 

#### 3.4.2. Irritation Test

##### Dermal Irritation Test

**Figure 6:**
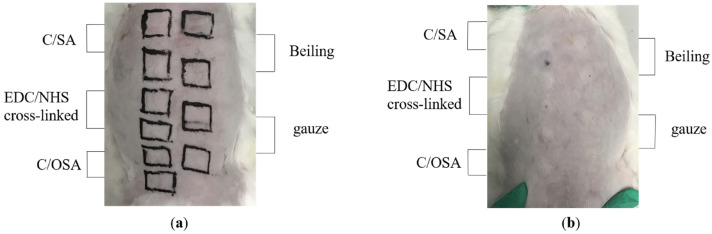
(**a**) Diagram illustrating the contact regions of dressings. (**b**) Positions for injecting the leach liquor.

Three New Zealand white rabbits were maintained under controlled conditions, with a constant temperature of 25 ± 1 °C, access to food and water ad libitum, and a 12 h dark/light cycle. Prior to the experiment, all rabbits were anesthetized with 3% pentobarbital sodium (30 mg/kg) via the ear vein. After anesthesia, the dorsal side of the rabbits was de-haired and sterilized using 75% ethanol. All samples were cut into approximately 2.25 cm^2^ (1.5 × 1.5 cm) under sterile conditions. They were then moistened with sterile water and affixed to the designated region on the dorsal side (as depicted in Figure 6a) for a duration of 4 h. Gauze was employed as a blank control. Following the removal of dressings, the examination areas were designated with a non-irritating permanent marker, washed with lukewarm water at 37 °C, and dried. The grading of erythema, crusting, and edema was recorded at 24, 48, and 72 h for all rabbit test and control sites, following the guidelines of ISO 10993-10: 2021 (E) [67]. The total skin reaction score and primary irritation index were calculated. To assess the potential for human irritation, the primary irritation index (PII) was calculated as the average score obtained from three rabbits. The PII was indicative of the level of irritation as follows: scores ranging from 0 to 0.4 denoted very mild irritation; scores ranging from 0.5 to 1.9 denoted slight irritation; scores ranging from 2 to 4.9 denoted moderate irritation; and scores ranging from 5 to 8 denoted severe irritation.

##### Intradermal Reaction Test

According to Figure 6b, intradermal reaction tests were conducted on three New Zealand great white rabbits. After administering anesthesia, the rabbits were intradermally injected on their backs with 0.2 mL of saline and leach liquor. The leach liquor was prepared according to the method outlined in Section 3.4.1. To serve as a control, normal saline was utilized. The occurrence and severity of local and peripheral skin erythema, scabs, and edema were promptly assessed and documented post-injection, as well as at 24, 48, and 72 h after injection. The PII was evaluated following the procedure outlined in the section of dermal irritation test.

##### Sensitization Test

The C/SA-based dressings were evaluated for their potential to cause delayed dermal contact sensitization using a method that adhered to the requirements of ISO 10993-10: 2021 (E) [67].

Fifty guinea pigs were weighed and randomly divided into five groups: a C/SA group, an EDC/NHS cross-linked group, a C/OSA group, a Beiling group, and a gauze group. The test samples were prepared in normal saline, and reagent controls (normal saline without test samples) were also prepared. The configuration ratios are described in Section 3.4.1. The fur on the back of each animal was removed with a clipper 24 h prior to the test, and the skin was sterilized using 75% ethanol. The experiment consisted of three phases: induction, local patch, and stimulation. In the first phase, each extract was intradermally injected into 10 test guinea pigs. In the second phase, one week after the intradermal injection, the back hair was shaved again, and the skin was sterilized. To induce sensitization, a filter paper measuring 2 × 4 cm, soaked in the leachate of the experimental group (saline for the control group), was applied to the injection sites of 10 test guinea pigs and fixed for 48 h. After a period of recovery, the trial progressed to the third phase. After a minimum of 14 days of localized patching, hair on the abdomen was shaved and sterilized. The filter paper was then submerged in the leachate and attached to the abdomen for 24 h. At 24, 48, and 72 h after removing the patch, all sites were evaluated, and any reactions such as skin erythema, scab formation, or edema were documented. The rules for scoring are described in the section dedicated to the dermal irritation test.

#### 3.4.3. Cytotoxicity In Vitro

The first requirement for biomedical materials is that they should not have any toxic effects on the human body. To evaluate cytotoxicity, we conducted a test in accordance with ISO 10993-5: 2009 (E): Biological evaluation of medical devices [56]. The MTT reduction assay utilized L929 cells (NCTC clone 929, ATCC). These cells were cultured in DMEM medium supplemented with 10% (*v*/*v*) FBS and 1% (*v*/*v*) antibiotics (penicillin and streptomycin) at a temperature of 37 °C in an environment with 5% CO_2_. C/SA-based dressing samples with the same size as a 12-well plate were produced and sterilized with ^60^Co for 18 h with an accumulative dose of 20 kGy. Commodities and gauze were also cut to the same size and sterilized under the same conditions. Subsequently, the samples were pre-wetted with DMEM medium and placed in a 37 °C, 5% CO_2_ incubator (HF90, Shanghai, China) for 24 h, and then the medium was removed. Fibroblast suspension was inoculated onto each sample at a density of 2 × 10^4^ cells per well and incubated for 48 h at 37 °C with 5% CO_2_ in a humidified incubator. After discarding the old medium, the samples were washed with DPBS and replaced with fresh medium containing 200 μL of MTT solution. The samples were then incubated for 4 h, and the absorbance of the resulting solution was measured at 490 nm using an enzyme marker (Metash, Shanghai, China). Cells cultured in dressing-free medium were used as the control. The relative growth rate (RGR) of the cells was calculated as the average of three parallel experiments according to the following formula:

(1) $$RGR(\%)=({Abs}_{s}/{Abs}_{c})\times 100$$

where *Abs*_s_ represents the absorbance values of the samples, and *Abs*_c_ represents the absorbance values of the blank control.

### 3.5. In Vivo Model of Femoral Artery Hemorrhage

The study aimed to evaluate the hemostatic effect of C/SA-based dressings using a rat femoral artery injury model [58,68]. Forty-five Wistar rats were randomly assigned to 5 groups. During the experiment, the rats were anesthetized with 3% sodium pentobarbital (30 mg/kg) administered intravenously. The femoral artery was carefully isolated from the surrounding muscle tissue, veins, and nerves. Subsequently, the femoral artery was exposed and clamped with forceps at a specific distance from the right side of the thigh. An incision was then made to induce bleeding from the injury site. Blood immediately flowed out of the wound and was wiped away with cotton wool after approximately 5 s. Hemostatic materials (C/SA, EDC/NHC cross-linked C/SA, and C/OSA) were pressed onto the bleeding wound with vertical pressure weighing 100 g. Sterile gauze and commercially available dressings were used as blank and positive controls, respectively. After 60 s of hemostasis, the dressing was removed and observed every 20 s until successful hemostasis was achieved. The bleeding time, blood loss, and success rate of hemostasis were recorded and calculated. The experiment was also documented through photographs.

### 3.6. Statistical Analysis

Unless otherwise specified, each sample was measured in three replicates. The average ± standard deviation was used to present all quantitative findings. The statistical analysis involved conducting analysis of variance (ANOVA) alongside Tukey’s multiple comparison test to determine significance (*p* < 0.05).

## 4. Conclusions

Porous sponge hemostatic dressings were prepared using Nile tilapia skin collagen and SA. They were cross-linked separately by EDC/NHS and OSA and compared with commercially available products and gauze. The experimental results demonstrated a 100% success rate of hemostasis in the rat femoral artery hemorrhage model, with control bleeding. When the mass ratio of SA to collagen was 1:5, the blood clotting time of the rat femoral artery in the dressings was approximately half shorter than that of the control group and significantly lower than that of the commercial hemostatic dressing group. Additionally, the mass ratio of dressings to the blood loss ranged from 1:13 to 1:16, which was significantly lower than the control group. These results suggested that the prepared dressings were effective for wounds characterized by acute massive blood loss. Furthermore, research indicated that the porous C/SA sponge could be applied to post-traumatic or exuding wounds (ulcers), and it demonstrated superior performance compared to previous studies on chitosan. Biocompatibility evaluation studies indicated that the C/SA-based dressings had good cytocompatibility and could promote cell proliferation. Prior to applying the dressing to the skin to stop bleeding, it was important to conduct sensitization and irritation studies. The study results confirmed that the hemostatic dressing was non-dermal irritant and non-dermal sensitizing. In summary, this study demonstrated that the addition of SA to collagen and its crosslinking with EDC/NHS or OSA could enhance the performance of the dressing, making it beneficial for the biomedical field.

## Figures and Tables

**Table 1:** The physicochemical properties of C/SA-based dressings.

	C/SA	EDC/NHS Cross-Linked C/SA	C/OSA
Loss on drying (%)	16.30 ± 0.09	13.52 ± 0.42	16.77 ± 0.01
Total ash content (%)	9.17 ± 0.22	0.29 ± 0.02	6.99 ± 0.12
Carbodiimide residue (mg)	-	0	-

**Table 2:** Scoring of erythema, scar formation, and edema formation in the stimulation test of the New Zealand white rabbit and the sensitization test of the guinea pig.

	C/SA	EDC/NHS Cross-Linked C/SA	C/OSA	Beiling	Gauze
Dermal irritation test					
24 h	0	0	0	0	0
48 h	0	0	0	0	0
72 h	0	0	0	0	0
PII	0	0	0	0	0
Intradermal reaction test					
24 h	0	0	0	0	0
48 h	0	0	0	0	0
72 h	0	0	0	0	0
PII	0	0	0	0	0
Sensitization test					
24 h	0	0	0	0	0
48 h	0	0	0	0	0
72 h	0	0	0	0	0

**Table 3:** Evaluation of hemostatic efficacy in a femoral artery bleeding model in vivo.

	Blood Clotting Time (s)	Blood Loss (g)	Success Rate of Hemostasis (%)
C/SA	140 ± 8.94 ^a^	1.24 ± 0.04 ^a^	100
EDC/NHS cross-linked	132.5 ± 12.82 ^a^	1.11 ± 0.07 ^a^	100
C/OSA	138.75 ± 8.35 ^a^	1.03 ± 0.08 ^a^	100
Beiling	165.71 ± 12.72 ^b^	1.38 ± 0.07 ^a^	100
Gauze	251.43 ± 10.69 ^c^	1.78 ± 0.08 ^b^	0

Note: Distinct lowercase letters represent significant difference (*p* < 0.05).

## Data Availability

The datasets generated and analyzed during the current study are available from the corresponding authors upon reasonable request.
